# Impacts of floods on dysentery in Xinxiang city, China, during 2004–2010: a time-series Poisson analysis

**DOI:** 10.3402/gha.v7.23904

**Published:** 2014-08-05

**Authors:** Wei Ni, Guoyong Ding, Yifei Li, Hongkai Li, Baofa Jiang

**Affiliations:** 1 Department of Epidemiology and Health Statistics, School of Public Health, Shandong University, Jinan, People's Republic of China; 2 Shandong University Climate Change and Health Center, Jinan, People's Republic of China; 3 Department of Occupational and Environmental Health, School of Public Health, Taishan Medical College, Taian, People's Republic of China

**Keywords:** floods, dysentery, Poisson regression, longitudinal analysis, relative risk

## Abstract

**Background:**

Xinxiang, a city in Henan Province, suffered from frequent floods due to persistent and heavy precipitation from 2004 to 2010. In the same period, dysentery was a common public health problem in Xinxiang, with the proportion of reported cases being the third highest among all the notified infectious diseases.

**Objectives:**

We focused on dysentery disease consequences of different degrees of floods and examined the association between floods and the morbidity of dysentery on the basis of longitudinal data during the study period.

**Design:**

A time-series Poisson regression model was conducted to examine the relationship between 10 times different degrees of floods and the monthly morbidity of dysentery from 2004 to 2010 in Xinxiang. Relative risks (RRs) of moderate and severe floods on the morbidity of dysentery were calculated in this paper. In addition, we estimated the attributable contributions of moderate and severe floods to the morbidity of dysentery.

**Results:**

A total of 7591 cases of dysentery were notified in Xinxiang during the study period. The effect of floods on dysentery was shown with a 0-month lag. Regression analysis showed that the risk of moderate and severe floods on the morbidity of dysentery was 1.55 (95% CI: 1.42–1.670) and 1.74 (95% CI: 1.56–1.94), respectively. The attributable risk proportions (ARPs) of moderate and severe floods to the morbidity of dysentery were 35.53 and 42.48%, respectively.

**Conclusions:**

This study confirms that floods have significantly increased the risk of dysentery in the study area. In addition, severe floods have a higher proportional contribution to the morbidity of dysentery than moderate floods. Public health action should be taken to avoid and control a potential risk of dysentery epidemics after floods.

Dysentery, caused by *Shigella* species (bacillary dysentery) or *Entamoeba histolytica* (amebic dysentery), remains a major public health problem around the world, especially in some developing countries (1). In China, despite the improvement of public health management for diseases control and prevention, dysentery disease still has a higher relapse rate, which seriously endangers public health (2). The incidence of dysentery each year ranged from 16.38 to 40.14 per 100,000 during 2004–2010 in Henan Province, which was relatively high compared with other provinces in China (3). Xinxiang, one of the northern cities of Henan Province, confronted the public health problem with a proportion of reported cases of dysentery being the third highest among the notified infectious diseases during 2005–2009 (4).

Due to climate change, heavy precipitation is expected to increase the occurrence and magnitude of extreme flood events, recognized as the most frequent and devastating type of natural disaster in the world (5). During 2004–2010, Xinxiang suffered from frequent floods due to persistent and heavy precipitation, which were responsible for many victims of natural disasters and economic losses (6 –10). Some studies suggested that floods due to heavy precipitation may affect the morbidity and mortality of dysentery (11–13). After floods, the morbidity of dysentery may be increased by the transmission and infection of the pathogens (14, 15). However, the association between floods and dysentery is far from clear. After fully controlling for pre-flood rate differences and seasonality, there was no clear evidence of excesses found in dysentery risk during or after floods (16). An investigation following massive flooding found no evidence of outbreaks or increased levels of gastrointestinal illness, and no mortality associated with gastrointestinal symptoms was identified (17). Above all the studies analyzing the association between floods and dysentery, they were only conducted in terms of one single flood event, with a lack of a longitudinal analysis. To our knowledge, there has been no research quantifying the risk of dysentery after a few floods in one region. In addition, with little research conducted in China, the effects of different degrees of floods on dysentery disease remains unknown.

In order to understand the risk of dysentery because of floods in Xinxiang during 2004–2010, our study aims to explore and quantify the possible relationship between different degrees of floods and the morbidity of dysentery, based on a long-term disease surveillance and floods data. Results will be able to predict the dynamics of dysentery epidemics by a best-fit Poisson regression model and contribute to a better understanding of the health impacts of flooding and assist in local strategies to prevent and reduce the risk of infectious diseases associated with floods.

## Materials and methods

### Study area

**Fig. 1:**
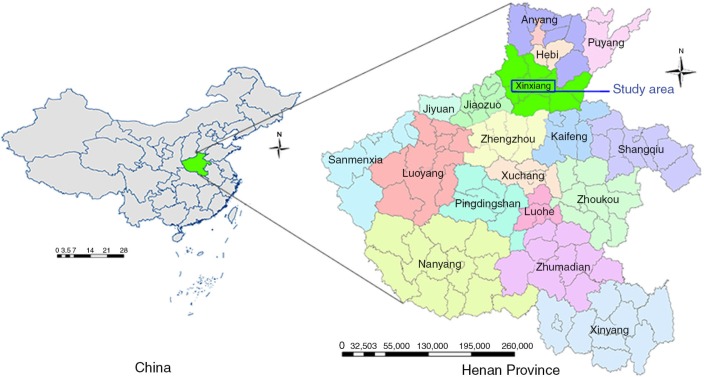
Location of Xinxiang in Henan Province, China.

Figure 1 shows the geographic position of Xinxiang city in the north of Henan Province, which is situated in the middle reaches of the Yellow River. It is located between latitude 34°55′–35°50′N and longitude 113°30′–115°30′E, which means that the city has a warm temperate continental monsoon climate with an average annual temperature of 13.9–14.6°C, with average rainfall per year ranging from 580 to 640 mm (18). The drinking water in Xinxiang includes well water, water supplied through pipes, and various purified waters. Xinxiang is an agricultural city, wherein the proportion of rural population is markedly high with poor health habits and hygiene.

### Data collection and management

#### Disease surveillance data

Monthly disease surveillance data on dysentery from 2004 January to 2010 December were obtained from the Notifiable Diseases Surveillance System (NDSS). All dysentery cases were defined based on the diagnostic criteria and principles of management for dysentery (GB 16002-1995) issued by Ministry of Health of China. According to the National Communicable Disease Control Act, physicians in hospitals must report every case of dysentery to the local health authority. Then, the local health authority must report these cases to the next level of the organization within 24 h (19). Therefore, it is believed that the degree of compliance in disease notification over the study period was consistent.

#### Data on floods

The yearbooks of meteorological disasters in China recorded the occurrence, deaths, damage area, and economic loss of floods in detail from 2004 to 2010 (20). The definition of flooding, according to the yearbooks of meteorological disasters in China, is a natural disaster resulting from the rivers overflowing due to short-term heavy precipitation, which leads to farmland and cities submerged, casualties and economic losses. A flooding causing death tolls between 10 and 30 or the economic losses between 1 and 3 billion RMB (about 16–48 million dollars) is classified as moderate flooding. One severe flooding is classified as the flooding causing more than 30 deaths or more than 3 billion RMB (about 48 billion dollars) economic losses. According to the yearbook of meteorological disasters in China, there were 10 flood events recorded in Xinxiang city from 2004 to 2010, including six episodes of moderate floods and four episodes of severe floods.

Flooding per se would be a variable depending on the quantitation over a shorter time period than a month. But in our study, we analyzed monthly data to assess the effects of floods on the dysentery disease based on time-series data from 2004 to 2010 which included flooded, non-flooded, pre-flooded and post-flooded months, and the same period over other years, so that monthly data would estimate the effects of floods well.

#### Demographic and meteorological data

Demographic data for Xinxiang was obtained from the Center for Public Health Science Data in China (http://www.phsciencedata.cn/). Monthly meteorological data were obtained from the China Meteorological Data Sharing Service System (http://cdc.cma.gov.cn/). The meteorological variables included monthly cumulative precipitation (MCP), monthly numbers of days with precipitation (MNDP), monthly average wind velocity (MAWV), monthly average temperature (MAT), monthly average air pressure (MAAP), and monthly average relative humidity (MARH).

### Statistical analysis

First, a descriptive analysis was performed to describe the distribution of dysentery morbidity and meteorological factors between the flooded and non-flooded months through the Wilcoxon-rank sum test. According to the reproducing of pathogens and the latency of dysentery disease, a time lag of 0–2 months was considered in this study (21). Spearman's correlation was used to examine the association between the monthly morbidity of dysentery, floods, and climate factors with various lagged values. The lagged value with the maximum correlation coefficient for each variable was selected for inclusion in the subsequent analysis.

Poisson regression, using a generalized linear model (GLM), was used to quantify the association between floods and the morbidity of dysentery adjusted for seasonality, secular trend, and lagged effects. In Poisson regression, the dependent variable is assumed to follow the Poisson distribution. In general, the multiple Poisson regression model can be written as (22):

$$\ln(Y)=\beta_{0}+\beta_{1}X_{1}+\beta_{2}X_{2}+...$$

Time-series Poisson regression models have been applied in several epidemiological studies to explore the relationship between climatic variables and infectious diseases (23, 24). In this study, the time-series Poisson regression model was, from the first time, applied to analyze a multiple categorical variable defined by non-flood, moderate, and severe floods to estimate the risks of different degrees of floods on morbidity of dysentery. Some studies indicated that temperature, precipitation, relative humidity, wind velocity, and air pressure have significant effects on the morbidity of dysentery (25–28). Thus, we fixed the floods, monthly number of days with flooding, and the seasonal variables in this model, determining other meteorological variables through a process of manually entering and omitting variables from the model in a stepwise manner with the regulation for elimination being a *p*>0.05 in order to adjust and further estimate the effect of floods on the monthly morbidity of dysentery. The regression model was described as follows:

$$\ln(Y_{t})=\beta_{o}+\beta_{1}t+\beta_{2}\sin(2\pi t/12)+\beta_{3}\text{flood}+\beta_{4}\text{flood duration}+\beta_{i}X_{\text{i}}(t-n)$$

where *Y*_*t*_ denoted the monthly morbidity of dysentery at time *t*. The coefficients were individually represented by *β*_*o*_ through *β*_*i*_. *X*_*i*_ was the monthly data of meteorological variables selected into the model. The different degrees of floods, namely non-flood, moderate floods, and severe floods were represented by 0, 1, and 2, respectively, and flood duration represented the monthly number of days with flooding. The term *t–n* in the subscript represented the n-month lagged time. This model included lagged values which were used to control the autocorrelation of explanatory variables. With consideration of seasonality and long-term trend, the proposed model included a triangular function, sin (2*πt*/12), to reveal the seasonal component in series.

In order to estimate the attributable contribution of floods to the morbidity of dysentery, the regression coefficients of different degrees of floods were transformed using the equation: $ARP=\frac{100^{}(e^{\alpha }-1)}{e^{\alpha }}$ (24).

This equation estimated the attributable risk proportion (ARP) of floods to the infection of dysentery, which revealed the percent change in morbidity associated with different types of floods. The statistical analysis was performed using SPSS 16.0 (SPSS Inc., USA) and software R 2.3.1 (MathSoft Inc., USA).

## Results

### Descriptive analysis for the disease and meteorological data

**Fig. 2:**
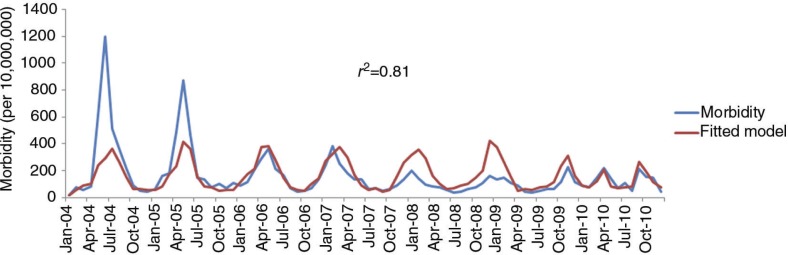
Dynamics of dysentery in Xinxiang with the analysis of Poisson regression model from 2004 to 2010 (Morbidity per 10,000,000 population).

A total of 7591 cases of dysentery were notified in the study area over non-flooded and flooded months from 2004 to 2010. Table 1 displays a summary of the distribution of morbidity of dysentery and meteorological variables by month in the Xinxiang city. The morbidity of dysentery, MCP, MNDP, MAT, MAAP, and MARH were significantly different between flooded and non-flooded months (*p*<0.05), which indicated that the climatic conditions differed between the months with or without floods. Figure 2 shows the distribution of monthly morbidity of dysentery in Xinxiang, with a trend of declining over the study period. Moreover, more dysentery cases occurred in summer and autumn in Xinxiang (Table 1).

**Table 1:** Description of dysentery morbidity and climate variables from 2004 to 2010 in Xinxiang city

Analyzing variables	Flooded months	Mean±SD	Min	P_25_	Median	P_75_	Max
Morbidity of dysentery	No	159±153	19	66	105	164	1,200
	Yes*	173±185	34	61	126	250	511
MCP (*mm*)	No	27.4±31.7	0	3.8	13.2	39.7	138.2
	Yes*	179.0±58.1	112.5	145.3	157.8	213.3	213.3
MNDP (days)	No	5±3	0	2	5	7.25	14
	Yes*	12±2	10	11	12.5	14	15
MAWV (m/s)	No	2.1±0.5	1.1	1.7	2.1	2.5	3.4
	Yes	1.9±0.3	1.5	1.7	1.8	2.1	2.4
MAT (°C)	No	13.7±9.3	−1.2	4.5	15	21.7	28.3
	Yes*	25.7±2.4	21.5	24.1	26.6	27.1	28.6
MAAP (hPa)	No	1009.0±8.0	993.3	1001.7	1010.6	1015.9	1022.2
	Yes*	998.3±4.6	994.4	995.0	995.9	1001.4	1006.9
MARH (%)	No	60.7±9.2	35.0	54.8	61.0	68.0	77.0
	Yes*	78.8±2.9	74.0	77.3	79.0	80.5	84.0

SD=standard deviation; Min=minimum; P_25_=the 25th percentile; P_75_=the 75th percentile; Max=maximum; MCP=monthly cumulative precipitation; MNDP=the monthly number of days with precipitation (≥0.1*mm*); MAWV=monthly average wind velocity; MAT=monthly average temperature; MAAP=monthly average air pressure; MRH=monthly relative humidity.

* *p*<0.05 vs. non-flooded month.

### Spearman's correlation analysis

Table 2 shows the results of Spearman's correlation test conducted to determine the relationship between the morbidity of dysentery and explanatory variables during the study periods. The results indicated that moderate and severe floods were positively correlated to the monthly morbidity of dysentery with no month lagged (*r*=0.24, *p*=0.03). The monthly morbidity of dysentery was positively correlated with monthly numbers of days with flooding, MCP, MNDP, MAT and MARH, but the morbidity was negatively correlated with MAAP. The lagged effects of all these climatic variables were incorporated in the next regression analysis.

**Table 2:** Correlations between the morbidity of dysentery and explanatory variables among monthly data in Xinxiang from 2004 to 2010

Monthly climate variables	Lag (months)	*r*	*p*
Moderate and severe floods	0	0.24	0.03
	1	0.21	0.06
	2	0.19	0.10
The number of days with floods	0	0.32	0.01
	1	0.28	0.09
	2	0.22	0.12
Cumulative precipitation	0	0.65	<0.01
	1	0.64	<0.01
	2	0.52	<0.01
The number of days with precipitation	0	0.63	<0.01
	1	0.58	<0.01
	2	0.43	<0.01
Average wind velocity	0	−0.34	<0.01
	1	0.32	0.08
	2	0.02	0.23
Average temperature	0	0.77	<0.01
	1	0.82	<0.01
	2	0.67	<0.01
Average air pressure	0	−0.68	<0.01
	1	−0.79	<0.01
	2	−0.73	<0.01
Average relative humidity	0	0.58	<0.01
	1	0.32	<0.01
	2	0.04	0.73

### Regression analysis

A Poisson regression model was conducted to determine the relationship between monthly morbidity of dysentery and different degrees of floods controlled for climatic variables listed in Table 3, and all the explanatory variables with appropriate lag month were significantly included in the model. Figure 2 shows that the dynamic of the monthly morbidity of dysentery corresponded well with this regression model during the study period. It demonstrated that the goodness-of-fit with a correlation between observed and expected monthly morbidity of dysentery was 81% (*r* ^2^=0.81). Table 3 shows the parameters from the Poisson regression model. The results indicated that both moderate and severe floods had statistically significant effects on the incidence of dysentery. After controlling for climatic factors, the monthly morbidity of dysentery was positively correlated with moderate floods (RR: 1.55, 95% CI =1.42–1.70) and severe floods (RR: 1.74, 95% CI=1.56–1.94).

**Table 3:** Parameters from Poisson regression model for dysentery diseases*

Variables	Coefficients (95% CI)	p*	RR (95% CI)
Intercept	23.13 (11.65, 34.61)	<0.01	–
T (month)	0.06 (0.04, 0.07)	<0.01	–
sin(2*πt*/12)	0.05 (0.02, 0.08)	<0.01	–
Moderate floods	0.44 (0.35, 0.53)	<0.01	1.55 (1.42,1.70)
Severe floods	0.55 (0.44, 0.66)	<0.01	1.74 (1.56,1.94)
Flood duration	−0.03 (−0.05, −0.001)	<0.01	–
MCP	0.0001 (0.0007, 0.0009)	<0.01	–
MNDP	0.03 (0.02, 0.04)	<0.01	–
MAWV	−0.036 (−0.040, −0.031)	<0.01	–
MAT	0.04 (0.03, 0.05)	<0.01	**–**
MAAP	−0.02 (−0.03, −0.01)	<0.01	–
MARH	0.010 (0.006, 0.013)	<0.01	–

* R square of the model was 0.81.

Flood duration=the monthly number of days with flood. MCP=monthly cumulative precipitation; MNDP=the monthly number of days with precipitation (≥0.1*mm*); MAWV=monthly average wind velocity; MAT=monthly average temperature; MAAP=monthly average air pressure; MRH=monthly relative humidity.

* *p*<0.05 vs. non-flooded month.

Based on the estimates of parameters from the Poisson regression model and the formula of ARP discussed earlier, ARPs of moderate floods and severe floods to the monthly morbidity of dysentery were 35.53 and 42.48%, respectively.

## Discussion

This is the first study to quantify the association between different degrees of floods and dysentery diseases in Xinxiang, China. It confirmed that floods will bring more morbidity of dysentery following flooding based on the analysis of 10 times floods including six episodes of moderate floods and four episodes of severe floods from 2004 to 2010. Results indicate that different degrees of floods have significantly influenced the dynamic of dysentery, revealing a relationship between the time-lag effect, long-term trend, and other meteorological variables.

The results from this study indicate that floods could significantly increase the risk of dysentery in Xinxiang. Some studies reported an increased risk of infectious diarrhea following floods in both developing countries (29–31) and developed countries. For example, in developed countries, flooded households were significantly associated with a greater risk of diarrhea than non-flooded homes during the 2001 floods in Texas (OR: 10.8, *p*<0.01) (32). In the town of Lewes in Southern England, flooding of houses was significantly associated with increased risk of gastroenteritis (RR: 1.7, *p*<0.05) (33). In addition, another study from the United States revealed that an increase in the incidence of diarrhea during the floods was observed (RR: 1.29, 95% CI: 1.06–1.58), and this effect was pronounced among persons with potential vulnerability to infectious diarrhea (34). In Germany, a study showed that the major risk factor for diarrhea was contact with floodwater (OR: 5.8, 95% CI: 1.3–25.1) (35). However, similar findings have been reported in our study. Our study shows that the risk for the morbidity of dysentery following moderate floods was lower than that following severe floods. It provides evidence that different degrees of floods can be relevant to the occurrence of dysentery. Heavy rainfall may cause flooding and change in the living environment. Some studies showed that water sources for drinking and recreation, because of flooding after extreme precipitation, have been associated with water-borne disease outbreaks and epidemics such as dysentery 36–42 . During the flood period, local water quality can be seriously compromised via diverse means, a significant one being the cross-contamination of water sources due to infiltration and inflow between sewage and water pipes (12, 43, 44). During the beginning of the flood period after heavy precipitation, the pathogens could grow fast and reproduce rapidly under a suitable environment, and then may spread through the contaminated water or food (13). With the increase of precipitation, floods may affect the local water sources and supply systems, as well as sewerage and waste-disposal systems, and other health infrastructures. Consequently, flooding probably washed contamination into water sources to increasing the transmission of enteric pathogens during severe floods (45), causing the deterioration in source water quality and more opportunities for individuals to have contact with floodwater.

In China, contaminated water may not be a direct risk factor for dysentery after floods because of a tradition of drinking boiled water. However, water is a significant component of many foods and it can be added directly as an ingredient or be present as part of the raw materials, which means that the contaminated drinking water can be closely associated with food following floods. The main pathways through which water can contaminate the food products in a factory include the incoming water by itself, the factory environment including the water storage and the distribution system, and factory workers (46). Food producers legitimately add water to food when processing starts. During the process of food production, water is usually used as a processing aid including as a thermal transfer medium, as a transport medium, and for cleaning. Due to contaminated water used in these processes, the contamination of food products during transformation can play an important role in the transmission of pathogens, leading to an increase in the risk of dysentery infection. A study conducted in Xinxiang showed that the health habits of food processing workers were poor with a low level of food safety knowledge, and their attention to food hygiene was inadequate (47), which could be a hazard for the spread of dysentery through contaminated foods after floods.

In addition, Henan Province where Xinxiang is located is an agricultural province with a large rural population. Food may be contaminated by floodwater through the poor health habits of farmers, such as cleaning plates and fruits using contaminated water directly. The food coming into contact with contaminated water is likely to increase the opportunities for transmission of dysentery following floods. People often in contact with pathogens neglect the potential risk for the transmission of communicable diseases. It is expected that recreation in floodwater, such as swimming in contaminated surface waters after floods, could spread the infection and increase the risk of dysentery too. After severe flood events with more serious damage and losses, the potential risk factors mentioned above may bring more dysentery cases than the moderate flood.

This study has identified a larger attributable contribution of severe floods to dysentery in the population than moderate floods, which suggests that the degree of increased incidence due to dysentery caused by severe floods is relatively more severe than that by moderate floods. It revealed the intensity of floods impacting on the dysentery and provided implication of public health and disease prevention to the disease prevention department. Public health action (e.g. water, waste, and die management; attention to personal hygiene; and conducting surveillance) should be taken during the flood period, which will result in preventing a dysentery epidemic.

Limitations of this study should be acknowledged. One of the main limitations is that there are many factors that can affect the transmission of dysentery, for example, human activities, socioeconomic status, availability of health services, environmental hygiene, which could not be included in this analysis. Second, it is realized that weekly data could make the estimation of lagged time more accurate than monthly data. However, it is difficult to evaluate the exact lag time between dysentery and floods. Considering the risk factors on dysentery after floods, such as water source contaminated, food production processes, and recreation with floodwater, the lagged time for dysentery infection may be much longer than the dysentery incubation period itself, which is less than a month. Obviously, more studies with different socioeconomic and possible risk factors should be conducted to have a better understanding of the health impact of floods based on more comprehensive data.

## Conclusion

In conclusion, floods can significantly increase the risks of dysentery in the study area. In addition, severe floods can contribute more ARP to dysentery than moderate floods. Our findings have significant implications for developing strategies to prevent and reduce the health impact of floods.
